# The Effect of Salmon Food-Derived DOM and Glacial Melting on Activity and Diversity of Free-Living Bacterioplankton in Chilean Patagonian Fjords

**DOI:** 10.3389/fmicb.2021.772900

**Published:** 2022-01-11

**Authors:** Paulina Montero, Marcelo H. Gutiérrez, Giovanni Daneri, Bárbara Jacob

**Affiliations:** ^1^Centro de Investigación en Ecosistemas de la Patagonia (CIEP), Coyhaique, Chile; ^2^Center for Oceanographic Research COPAS Sur-Austral and COPAS COASTAL, Universidad de Concepción, Concepción, Chile; ^3^Departamento de Oceanografía, Universidad de Concepción, Concepción, Chile

**Keywords:** salmon aquaculture, glacial melting, dissolved organic matter, heterotrophic activity, bacterioplankton community diversity

## Abstract

Fjord ecosystems cycle and export significant amounts of carbon and appear to be extremely sensitive to climate change and anthropogenic perturbations. To identify patterns of microbial responses to ongoing natural and human-derived changes in the fjords of Chilean Patagonia, we examined the effect of organic enrichment associated with salmon aquaculture and freshening produced by glacial melting on bacterial production (BP), extracellular enzymatic activity (EEA), and community diversity of free-living bacterioplankton. We assayed the effects of salmon food-derived dissolved organic matter (SF-DOM) and meltwaters through microcosm experiments containing waters from Puyuhuapi Fjord and the proglacial fjords of the Southern Patagonia Icefield, respectively. Rates of BP and EEA were 2 times higher in the presence of SF-DOM than in controls, whereas the addition of autochthonous organic matter derived from diatoms (D-DOM) resulted in rates of BP and EEA similar to those measured in the controls. The addition of SF-DOM also reduced species richness and abundance of a significant fraction of the representative taxa of bacterioplankton of Puyuhuapi Fjord. In the proglacial fjords, bacterioplankton diversity was reduced in areas more heavily influenced by meltwaters and was accompanied by moderate positive changes in BP and EEA. Our findings strongly suggest that SF-DOM is highly reactive, promoting enhanced rates of microbial activity while could be influencing the diversity of bacterioplankton communities in Patagonian fjords with a strong salmon farming activity. These findings challenge the traditional view of phytoplankton production as the primary source of labile DOM that fuels heterotrophic activity in coastal ecosystems impacted by anthropogenic organic enrichment. Given the intensive local production of salmon, we analyze the significance of this emerging source of rich “allochthonous” organic substrates for autotrophic/heterotrophic balance, carbon exportation, and hypoxia in Patagonian fjords. The effect of human DOM enrichment can be enhanced in proglacial fjords, where progressive glacial melting exerts additional selective pressure on bacterioplankton diversity.

## Introduction

Chilean Patagonia (41°–56°S) is one of the most extensive fjord regions in the world (Iriarte et al., 2014a), characterized by variable hydrobiological regimes associated with strong seasonal and latitudinal patterns of precipitation, river and meltwater discharges, and light availability (Aracena et al., 2011; Pantoja et al., 2011a and references therein). The region also supports high rates of primary production (Montero et al., 2011, 2017a,b; Jacob et al., 2014) and significant carbon fluxes (González et al., 2013, 2016). In fact, Patagonian fjords are considered to act both as a net sink of atmospheric CO_2_ (Torres et al., 2011) and a site of significant burial of sedimentary organic carbon (Sepúlveda et al., 2011; Smith et al., 2015). Due to their significance to global carbon fluxes and their vulnerability to anthropogenic and climatic pressure, fjord regions have recently been classified as Aquatic Critical Zones (Bianchi et al., 2020). These areas cycle and export significant amounts of carbon and other biogeochemically active elements (Bianchi et al., 2020), including natural and human-derived organic compounds (e.g., Robinson et al., 2005). Thus, along the land-ocean aquatic continuum of fjords, organic matter is composed of variable fractions of terrestrial, marine and anthropogenic components, as observed in the fjords of northern Patagonia (González et al., 2019). Chilean fjords are impacted by climate change (e.g., ice melting and glacial retreat, warming), and allochthonous inputs derived from anthropogenic activities (e.g., aquaculture, agriculture, forestry, and hydroelectricity) that alter their structure and functioning and pose a serious threat for ecosystem “health” (Iriarte, 2018). In these fjords, salmon farming is the principal aquaculture activity (Buschman et al., 2006), contributing significantly to the economy of Patagonia (Quiñones et al., 2019), but also with potentially deleterious environmental consequences (Buschman et al., 2006). One major threat relates to release of large quantities of waste materials (Quiñones et al., 2019) which now provide an emerging source of organic and inorganic substrates for biological activity within fjord ecosystems (Iriarte et al., 2014b). The organic fraction of these allochthonous inputs combined with high production of autochthonous organic matter by phytoplankton (Montero et al., 2011, 2017a,b), provides a heterogeneous cocktail of organic substrates available to be exploited by heterotrophic microbes.

Dissolved organic matter (DOM) represents the major substrate fueling the heterotrophic activity of marine microorganisms (Cole et al., 1982; Azam et al., 1983), with bacterioplankton (Bacteria and Archaea) one of the main groups consuming marine DOM (Azam, 1998; del Giorgio and Cole, 1998; Turley et al., 2000; Cuevas et al., 2004; Montero et al., 2007, 2011; Attermeyer et al., 2014). The bulk of the autochthonous DOM pool is ultimately derived from phytoplankton production (Azam and Malfatti, 2007; Buchan et al., 2014) which is therefore considered an important driver of bacterioplankton abundance, activity and diversity (Cole et al., 1988; Azam and Malfatti, 2007; Buchan et al., 2014). A range of anthropogenic activities have altered the fluxes and cycling of organic carbon in the coastal ocean (Bauer et al., 2013), with potential effects on the availability of organic substrates for growth of heterotrophic organisms. In fjord ecosystems, salmon farming supplies allochthonous dissolved substrates through dissolution of organic particles derived from feces and uneaten feed (Wang et al., 2012). This organic material is considered highly degradable (Nimptsch et al., 2015) and could provide organic substrates for heterotrophic microbial activity (Yoshikawa and Eguchi, 2013; Nimptsch et al., 2015; Kamjunke et al., 2017) that are degraded and respired at rates comparable to those of autochthonous organic material (Yoshikawa et al., 2012, 2017; Yoshikawa and Eguchi, 2013). Relative changes in the supply of dissolved organic substrates from various sources can influence the diversity of bacterioplankton communities by modifying niche availability and metabolic activity (Buchan et al., 2014; Blanchet et al., 2016; Hoikkala et al., 2016; Lucas et al., 2016). However, few data are available on the impact of the above anthropogenic organic substrates on the structure and activity of microbial heterotrophic communities. For instance, in Chilean fjords, most research effort on the effects of salmon farming wastes has focused on impacts of inorganic nutrient enrichment on eutrophication and phytoplankton dynamics (Iriarte et al., 2012, 2014b; Jensen, 2012; Olsen et al., 2014; Sanchez et al., 2019) and on the structure (Olsen et al., 2017) and activity of specific functional groups of the bacterial community (Elizondo-Patrone et al., 2015). The potential effects of organic waste from salmon aquaculture on activity and community structure of heterotrophic microbes has received little attention.

Salinity is one of the major environmental drivers of physicochemical and biological variability in coastal ecosystems, and influences the abundance, growth, physiology, activity and diversity of microorganisms (del Giorgio and Bouvier, 2002; Langenheder et al., 2003; Apple et al., 2008; Laghdass et al., 2010; Lindh et al., 2015). In fjord waters, including Patagonian fjords, salinity influence primary productivity (González et al., 2013; Piquet et al., 2014) and is a principal factor that controls microbial community structure (Gutiérrez et al., 2015, 2018; Balmonte et al., 2019). Freshwater discharge resulting from the melting of glaciers influences hydrography and circulation (Moffat, 2014), and its high load of suspended material and low concentrations of dissolved inorganic nutrients impact productivity, physiology and ecology of phytoplankton (Aracena et al., 2011; González et al., 2013; Piquet et al., 2014; Iriarte et al., 2018). Despite known effects of meltwaters on hydrobiological conditions, and evidence for transport of labile DOM by meltwaters (Hodson et al., 2008; Hood et al., 2009), few data are available on associated changes in heterotrophic activity and microbial diversity.

The effects of anthropogenic activity and climatic change in Patagonian fjord ecosystems have been the focus of considerable research in the last years (Iriarte, 2018) with evidence for significant retreat of numerous glaciers in the Patagonian Icefields (Rivera et al., 2012; Moffat, 2014 and references therein). Thus, in order to advance in identifying responses of microbial assemblages to ongoing natural and human-derived changes in environmental and trophic conditions of Patagonian fjords, we address here the question of how bacterial production, enzymatic hydrolysis and diversity of bacterioplankton communities respond to the organic enrichment associated with dissolved substrates derived from salmon aquaculture. We also examine freshening associated with meltwaters on the activity and diversity of heterotrophic microbial community in glacial fjords of the area of the Southern Patagonian Icefields. Our results will contribute to improve our current understanding of microbial processes, carbon fluxes and trophic status under shifting conditions in Chilean fjords.

## Materials and Methods

### Study Area and Sampling

**FIGURE 1:**
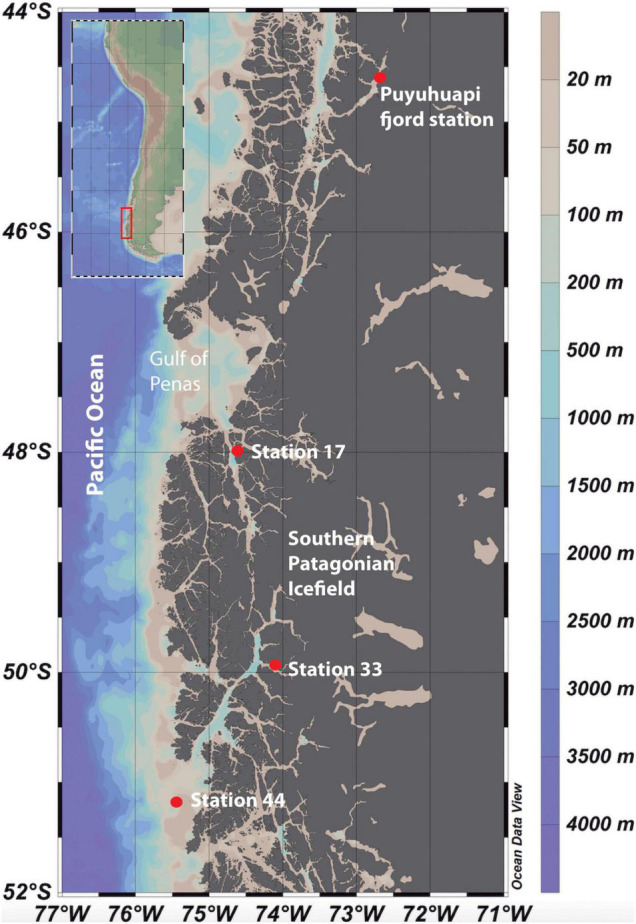
Study area within the fjords of Chilean Patagonia (Schlitzer, 2021). Inset shows the locations of the sampling stations in Puyuhuapi Fjord and in the fjord area of the Southern Patagonia Ice Field (Stations 17, 33, and 44).

Microcosm experiments were conducted to assay the effects of DOM derived from salmon food (SF-DOM) relative to autochthonous DOM (diatoms-derived organic substrates; D-DOM) on microbial heterotrophic activity and diversity. The assays were carried out using surface (2 m) and subsurface (20 m) waters collected with acid-cleaned Niskin bottles from a fixed station under no direct influence of salmon farming in Puyuhuapi Fjord (Figure 1). Sampling at the fixed station was conducted during five seasonal campaigns that encompassed two austral summers (March 2017, February 2019), one autumn (May 2018) and two winters (July 2018, July 2019) (Table 1). Puyuhuapi Fjord extends for about 90 km between 44°19′ S and 44°57′ S in northern Chilean Patagonia and connects with the coastal ocean through the Moraleda channel to the south, and the Jacaf Channel to the north (Schneider et al., 2014). The fjord is characterized by a two-layer estuarine type circulation with fresher waters in the top 5–10 m overlying saltier waters beneath. Freshwater is mainly provided by the Cisnes and Ventisquero rivers, precipitation and terrestrial runoff (Schneider et al., 2014), while deeper saline water originates from intrusions of subantarctic waters. Numerous salmon farms are present within the fjord, with 18 being active during the study periods (National Fisheries Services of Chile, SERNAPESCA, 2018).

**TABLE 1:** Variables measured during microcosm experiments with waters of Puyuhuapi and proglacial fjords.

Date	Station	Lat (°S)	Long (°W)	BP	EEA	BA	BB	BCC	DOC	BGE	DO	Inorganic Nutrients
*Puyuhuapi Fjord*												
March 2017	Fixed	44° 35′	72° 43′	√	√			√				
May 2018	Fixed	44° 35′	72° 43′	√	√			√	√			
July 2018	Fixed	44° 35′	72° 43′	√	√	√	√	√	√	√	√	
February 2019	Fixed	44° 35′	72° 43′	√	√	√	√	√	√	√	√	√
July 2019	Fixed	44° 35′	72° 43′	√	√	√	√	√				√
*Glacial fjords*												
November 2017	33	49° 56′	74° 9′	√	√			√	√			
	17	48° 02′	74° 38′	√	√			√	√			
	44	51° 12′	75° 29′	√	√			√	√			

*BP, bacterioplankton production; EEA, extracellular enzymatic activity; BA, bacterioplankton abundance; BB, bacterioplankton biomass; BCC, bacterioplankton community composition; DOC, dissolved organic carbon; BGE, bacterioplankton growth efficiency; DO, dissolved oxygen; inorganic nutrients, nitrate NO_3_ and phosphate PO_4_.*

The effect of meltwaters on microbial diversity and activity was assayed using microcosm transplant experiments carried out during the research cruise CIMAR 23 Fiordos in austral spring (November 2017) on board the R/V Cabo de Hornos (Figure 1). Surface and subsurface waters (2 and 20 m respectively) were collected with acid-cleaned Niskin bottles at three sampling stations: stations 17 and 33 have a strong glacial influence, with station 44 under stronger influence of oceanic waters (Figure 1 and Table 1). The Southern Patagonia Ice Field is the largest temperate ice field in the Southern Hemisphere, extending for about 370 km between 48°15′ S and 51°31′ S at an average longitude of 73°30′ W (Casassa et al., 2002). This ice field consists of 48 main basins and nearly 13,000 km^2^ of ice (Aniya et al., 1999). During recent years, frontal retreat and thinning have been evidenced in numerous glaciers in the region (Glasser et al., 2011; Rivera et al., 2012; Willis et al., 2012), with local fjords receiving considerable volumes of ice and meltwaters (Casassa et al., 2002). These meltwaters carry high loads of suspended sediments into the fjords, which attenuates light penetration into the water column (Jacob et al., 2014), and dilutes the concentration of silicic acid transported from other continental freshwater sources (Torres et al., 2014).

### Preparation of Dissolved Organic Matter Supplements

The diatom *Skeletonema pseudocostatum* was selected for the preparation of the autochthonous D-DOM supplement. *Skeletonema* is one of the main genera of phytoplankton in Puyuhuapi Fjord and often responsible for late winter blooms (Montero et al., 2017a). Axenic monocultures of *S. pseudocostatum* (strain CSA-15) were obtained from COPAS Sur-Austral strain collection (FICOLAB laboratory, University of Concepción). A liquid cell suspension from a mature culture of *S. pseudocostatum* was gently filtered through pre-combusted 0.7 μm glass fiber filters (Whatman GF/F) and the filtrate collected in a 1 liter pre-combusted glass bottle before being dispensed into the microcosms. For allochthonous SF-DOM, pellets of salmon food, provided by the Salmon Technological Institute (INTESAL, Chile), were milled, added to distilled water and stirred until a homogenous mixture was reached. To separate the dissolved fraction, the mixture was filtered through pre-combusted 0.7 μm glass fiber filters (Whatman GF/F) and the filtrates collected in a 1 liter pre-combusted glass bottle before adding to the microcosms. We estimated the carbon equivalent of the cell suspension based on cell density in the mature cultures and a conversion factor of 40 pg C cell^–1^ for diatoms from the coastal ocean off Chile (González, personal communication). Considering a carbon content of 0.1 g C per gram of salmon food utilized in this study (determined using a C/H/N analyzer LECO TruSpec^®^), we calculated the salmon food required to give a concentration of carbon equivalent to that provided by diatom cultures. The concentration of dissolved carbon in DOM supplements used in each experiment is shown in Table 2.

**TABLE 2:** Experimental conditions and derived parameters from microcosm incubations with waters of Puyuhuapi and proglacial fjords.

	Puyuhuapi Fjord											

Experimental conditions	Mar 17	May 18	Jul 18	Feb 19		Jul 19	Mar 17	May 18		Jul 18	Feb 19	Jul 19
**Control**	**Surface**						**Subsurface**					

ΔO_2(*tf–t*0)_ (mL L^–1^)			0.68	0.18								
BGE (%)			23	37						13	46	
DOC t0 (μM)		45 ± 5	72 ± 9	89 ± 5				35 ± 0		59 ± 15	72 ± 16	
DOC tf (μM)		16 ± 2	29 ± 8	38 ± 5				26 ± 3		33 ± 3	33 ± 5	
DOC_*consump*_ (%)		64	60	57				26		44	54	
NO_3_ t0 (μM)				0.2 ± 0.1		7.7 ± 1.6					9.9 ± 0.2	12.3 ± 0.3
NO_3_ tf (μM)				0.9 ± 0.2		9.5 ± 0.6					10.2 ± 0.8	11.6 ± 0.1
PO_4_ t0 (μM)				0.1 ± 0.0		0.8 ± 0.1					1.1 ± 0.2	1.5 ± 0.3
PO_4_ tf (μM)				0.1 ± 0.01		0.9 ± 0.04					1.1 ± 0.1	1.8 ± 0.1

**SF-DOM**	**Surface**						**Subsurface**					

DOC_*suppl*_ (μM)	92	90	82	233		90	92	90		82	233	90
ΔO_2(*tf–t*0)_ (mL L^–1^)			1.17	0.36								
BGE (%)			21	79						15	67	
DOC t0 (μM)		90 ± 0	104 ± 8	90 ± 11				113 ± 0		63 ± 19	84 ± 7	
DOC tf (μM)		24 ± 1	35 ± 8	41 ± 2				26 ± 5		27 ± 7	38 ± 6	
DOC_*consump*_ (%)		73	66	49				77		57	46	
NO_3_ t0 (μM)				0.2 ± 0.01		7.7 ± 2.4					11.1 ± 0.4	12.0 ± 3.7
NO_3_ tf (μM)				0.4 ± 0.02		8.5 ± 1.5					11.8 ± 2.2	12.5 ± 0.5
PO_4_ t0 (μM)				0.7 ± 0.1		1.0 ± 0.1					2.1 ± 0.2	1.6 ± 0.2
PO_4_ tf (μM)				0.8 ± 0.1		0.9 ± 0.1					1.9 ± 0.3	1.9 ± 0.01
BA × 10^6^ t0 (cells L^–1^)			738 ± 68	643 ± 19		661 ± 3				331 ± 1	942 ± 149	512 ± 48
BA × 10^6^ tf (cells L^–1^)			1761 ± 122	2605 ± 146		1084 ± 2				565 ± 11	2058 ± 160	447 ± 14

**D-DOM**	**Surface**						**Subsurface**					

DOC_*suppl*_ (μM)	87	138	100	187		82	87	138		100	187	82
ΔO_2(tf–t0)_ (mL L^–1^)			1.00	0.37								
BGE (%)			16	45						15	52	
DOC t0 (μM)		94 ± 15	103 ± 32	91 ± 15				105 ± 10		65 ± 11	72 ± 8	
DOC tf (μM)		25 ± 8	36 ± 1	42 ± 6				15 ± 4		31 ± 13	36 ± 6	
DOC_*consump*_ (%)		73	65	45				86		52	50	
NO_3_ t0 (μM)				0.2 ± 0.5		19.8 ± 5.2					11.9 ± 0.1	20.5 ± 2.9
NO_3_ tf (μM)				0.2 ± 0.01		18.7 ± 1.6					10.5 ± 0.4	26.8 ± 4.7
PO_4_ t0 (μM)				0.1 ± 0.01		3.1 ± 0.2					1.2 ± 0.3	3.4 ± 0.1
PO_4_ tf (μM)				0.2 ± 0.01		3.2 ± 0.1					1.1 ± 0.04	4.3 ± 0.03
BA × 10^6^ t0 (cells L^–1^)			696 ± 19	684 ± 106		803 ± 15				347 ± 8	696 ± 211	676 ± 6
BA × 10^6^ tf (cells L^–1^)			1975 ± 87	2858 ± 535		838 ± 2				371 ± 3	2161 ± 156	571 ± 16
**Proglacial fjord**	**Station 17**				**Station 33**				**Station 44**			
**Control**												
DOC t0 (μM)	103 ± 27				103 ± 18				203 ± 32			
DOC tf (μM)	75 ± 7				73 ± 8				125 ± 11			
DOC_*consump*_ (%)	27				29				38			
**Mixing**												
Δ Salinity	17.4				8.8				2.2			
DOC t0 (μM)	122 ± 1				91 ± 4				193 ± 11			
DOC tf (μM)	87 ± 5				88 ± 8				122 ± 21			
DOC_*consump*_ (%)	29				3				37			

*DOC, BA, and nutrient measurements are expressed as average ± SD.*

### Experimental Design of Microcosms in Puyuhuapi and Glacial Fjords

Water samples collected from 2 and 20 m depth in Puyuhuapi Fjord were sieved through a 22-μm mesh to remove large zooplankton and phytoplankton. Pre-sieved water was gently filtered through sterile membrane filters of 0.8-μm pore size (Millipore) using a peristaltic pump, in order to separate the free-living bacterioplankton fraction (<0.8 μm) and remove potential predators. The experimental design included the following treatments: (a) bacterioplankton + D-DOM, (b) bacterioplankton + SF-DOM, and (c) controls of bacterioplankton without addition of DOM supplements. Each microcosm treatment consisted of duplicate 10-L foil plastic bags with light protection (Supel™-Inert) incubated in a water bath mimicking *in situ* temperature. Water samples were collected at t0 and after 12, 24, 48, 72, and 96 (tf) hours during the incubations for chemical and microbiological parameters. The time elapsed between supplements addition and t0 measurements was approximately 1.5 h. Hydrographic conditions (temperature, salinity and dissolved oxygen) and inorganic nutrients concentrations in surface and subsurface waters from the study area are shown in Table 3.

**TABLE 3:** Environmental conditions in surface (sur) and subsurface (sub) waters at the different experimental periods in Puyuhuapi Fjord and in the sampling stations of the area of proglacial fjords.

	Puyuhuapi Fjord					Southern Ice Field		
	Mar	May	Jul	Feb	Jul		Nov 2017	
	2017	2018	2018	2019	2019	St. 17	St. 33	St. 44
S sur	15.21	23.64	29.45	20.75	18.21	13.82	21.80	30.87
S sub	30.93	30.03	31.53	30.61	31.50	31.19	30.64	33.06
T (°C) sur	15.45	10.44	9.35	16.20	7.49	10.75	10.86	10.92
T (°C) sub	12.29	11.14	10.47	12.07	10.59	9.55	3.82	9.05
DO sur (mL L^–1^)	n.d.	7.03	5.94	6.06	6.95	7.76	7.55	7.69
DO sub (mL L^–1^)	n.d.	4.97	4.51	4.47	4.70	6.04	5.26	6.33
NO_3_ (μM) sur	0.27	0.44	0.99	0.08	9.10	0.13	0.56	n.d.
NO_3_ (μM) sub	20.51	9.33	5.02	7.35	16.75	6.53	10.06	6.39
PO_4_ (μM) sur	0.27	0.45	0.38	0.17	0.89	0.02	0.53	n.d.
PO_4_ (μM) sub	1.75	1.20	1.05	0.84	1.87	0.89	1.29	0.9

*S, salinity; T, temperature; DO, dissolved oxygen; n.d., not determined.*

The influence of meltwaters was examined at the three stations located in the glacial fjord area. For each station 10-L duplicated microcosms were prepared using a 1:1 mixture of 0.8-μm filtered subsurface saline waters (i.e., bacterioplankton inoculum) and 0.2-μm filtered fresher surface waters. In addition, controls were prepared in 10-L microcosms with 0.8-μm filtered subsurface waters. Salinity differences between surface and subsurface waters were higher at stations 17 and 33 than at station 44 (Table 3). All microcosms were incubated on board in a water bath mimicking *in situ* temperature and were sampled at t0 and after 24, 48, and 72 (tf) hours for chemical and microbiological parameters. Hydrographic conditions (temperature, salinity and dissolved oxygen) and inorganic nutrients concentrations are shown in Table 3.

### Microbiological Analyses

Bacterioplankton production (BP) was estimated from incorporation of Leucine into proteins, using the centrifugation method (Smith and Azam, 1992). Samples from microcosms were taken at each sampling time, divided into three aliquots of 1.5 mL, and each incubated with L-[3,4,5-^3^H]-leucine (123.8 Ci mmol^–1^, 40 nM final concentration) in the dark for 1 h. A blank was prepared in the same way as samples, with the immediate addition of 100% trichloroacetic acid (TCA). After incubation, samples were extracted with 100% TCA, rinsed with 5% TCA and then centrifuged at 13,500 rpm twice for 15 min before removal of supernatant. One mL of liquid scintillation cocktail (Ecoscint; National Diagnostic) was added to each sample which were then counted for dpm using a Packard (Mod. 1600 TR) liquid scintillation counter. Leucine incorporation rates were transformed into bacterioplankton carbon following the procedure of Simon and Azam (1989). BP was calculated using a theoretical conversion factor of 1.5 kg C mol Leucine^–1^ (Simon and Azam, 1989). Bacterioplankton growth efficiency (BGE) was measured during campaigns in winter 2018 (July) and summer 2019 (February) and was calculated according to the following equation BGE = – (ΔBB/ΔDOC) (Eichinger et al., 2010), where ΔBB is the bacterioplankton biomass produced, and ΔDOC is the dissolved organic carbon consumed during the experiment. Over the same periods, dissolved oxygen (DO) concentration was measured during incubations using a fiber optical oxygen transmitter (Optical Oxygen meter FIBOX, PreSens^®^).

For extracellular enzymatic activity (EEA), duplicate 5 mL-aliquots of samples water from the microcosms at defined time intervals were incubated in the dark with L-leucine-4-methylcoumarinyl-7-amide (MCA-Leu) at 100 μM final concentration (Hoppe, 1983). Fluorescence was measured at time zero, and subsequently every ∼20 min for ca. 2 h at 365 nm excitation and 455 nm emission. Calibration curves were produced by measuring the fluorescence in water samples from each microcosm supplemented with the hydrolysis product MCA, at concentrations ranging between 0.03 and 0.5 μM. First order rate constants were calculated from the slope of the plot of ln (C_0_/(C_0_-P)) vs. time, where C_0_ is the initial concentration of the substrate MCA-leu and P is the concentration of the product MCA at time t (Pantoja and Lee, 1994). Hydrolysis rates were calculated by multiplying rate constants by C_0_. Discrete measurements of bacterioplankton production (BP) (Supplementary Figure 1) and extracellular enzymatic activity (EEA) (Supplementary Figure 2) were used to calculate the time-integrated rates during experimental period (96 and 72 h in Puyuhuapi and glacial fjords respectively) using the trapezoidal method.

Bacterioplankton abundance (BA) was determined using flow cytometry. Duplicate (1,350 μL) samples in cryovials were fixed using 150 μL of 1% glutaraldehyde, gently mixed and left in the dark at room temperature for 10 min before quick-freezing in liquid nitrogen and storage at –80°C. The samples were thawed at room temperature and stained with SYBR-Green I (4 μL) for 15 min in the dark and analyzed on a flow cytometer (InFlu^®^) equipped with a 488 nm laser in the Flow Cytometry Laboratory of the Millennium Institute of Oceanography, University of Concepción. Heterotrophic prokaryotes were detected using a 530 nm filter, and the intersections of SybrGreen vs. forward scatter (FSC) and SybrGreen vs. Chlorophyll were used to differentiate prokaryotes from other small fluorescent organisms. Bacterioplankton biomass was estimated using a conversion factor of 20 fg C per cell (Lee and Fuhrman, 1987).

For bacterioplankton community composition, 1-liter samples from t0 and tf from each treatment were filtered through 0.22 μm sterile membrane filters (Millipore) and stored at −20°C. DNA on filters was extracted using a PowerWater^®^ DNA Isolation Kit and cleaned using a Power Clean^®^ DNA Clean-up Kit (MOBIO Laboratories). Prokaryote 16S rRNA genes were amplified using primer set 515F (5′-GTGCCAGCMGCCGCGGTAA-3′) and 806R (5′-GGACTACHVGGGTWTCTAAT-3′). Amplification and sequencing were conducted on an Illumina MiSeq platform in commercial laboratories (Research and Testing Laboratory, Lubbock, TX, United States; Molecular Research DNA laboratory, MR DNA, Shallowater, TX, United States). The full MiSeq data set is available at the National Center for Biotechnology Information Sequence Read Archive (Accession numbers SRR15657559 to SRR15657658). Paired Illumina reads were processed using QIIME2 version 2020.2 (Bolyen et al., 2019). Quality filtering, denoising and chimera removal were carried out using the DADA2 method, and taxonomy of Amplicon Sequence Variants (ASVs) was assigned using the classify-sklearn function and a Naïve Bayes classifier trained against the Silva 132 99% reference sequences (Quast et al., 2013; Yilmaz et al., 2014; Bokulich et al., 2018). Analysis of beta and alpha diversity (measured as ASVs abundance) was estimated after removal of sequences identified as Chloroplast and after re-sampling with the rarefaction method using the minimum number of sequences per sample (4,951 and 16,951 for samples of Puyuhuapi and glacial fjord, respectively). We estimated similarity at the ASVs level based on the Bray-Curtis distance matrix index and conducted Principal Coordinate Analysis (PCoA) in R version 3.6.1 (R Core Team, 2019) using the package vegan (Oksanen et al., 2013). Statistical differences in the community composition between periods were tested using PERMANOVA analysis in R. ASVs heatmaps were produced in the Orange3 software (Demsar et al., 2013) in order to identify changes of the representative individual ASVs (representative ASVs defined as those containing more than 1,000 sequences) during incubations (from t0 to tf), and at tf in treatments relative to controls. Rarefaction curves for each prokaryote community were generated from the means of 10 randomized data sets in Qiime2.

In agreement with small to moderate samples size, non-parametric Mann-Whitney and Kruskal Wallis tests were utilized to test differences in BP, EEA, diversity, BA and DOC between control and treatments, depths and periods, and between times of incubations (t0 and tf). In order to look for common and specific patterns of response in the experiments, the dataset of activity and diversity was analyzed as a whole, as well as separated for different periods and/or depths.

### Dissolved Organic Carbon and Inorganic Nutrients

Water samples of 60-mL were collected for dissolved organic carbon (DOC) analysis from each microcosm at t0 and tf. Samples were filtered through pre-combusted (450°C, 6 h) 25-mm GF/F filters (∼0.7-μm) and then frozen at –20°C. Prior to analysis, the samples were acidified with 40 μL of phosphoric acid and decarbonated by purging with high purity CO_2_ free air (Cuevas et al., 2004). DOC concentration was determined using a catalytic high combustion TOC-5000 Shimadzu analyzer. Water samples of 500-mL were collected at t0 and tf for analysis of dissolved nitrate and phosphate from each microcosm. Samples were filtered through GF/F filters and the filtrates stored frozen at –20°C prior to analysis in the laboratory. Concentrations of these inorganic nutrients were determined spectrophotometrically according to standard methods (Strickland and Parsons, 1968).

## Results

### Bacterioplankton Production and Extracellular Enzymatic Activity During Dissolved Organic Matter Incubations in Puyuhuapi Fjord

**FIGURE 2:**
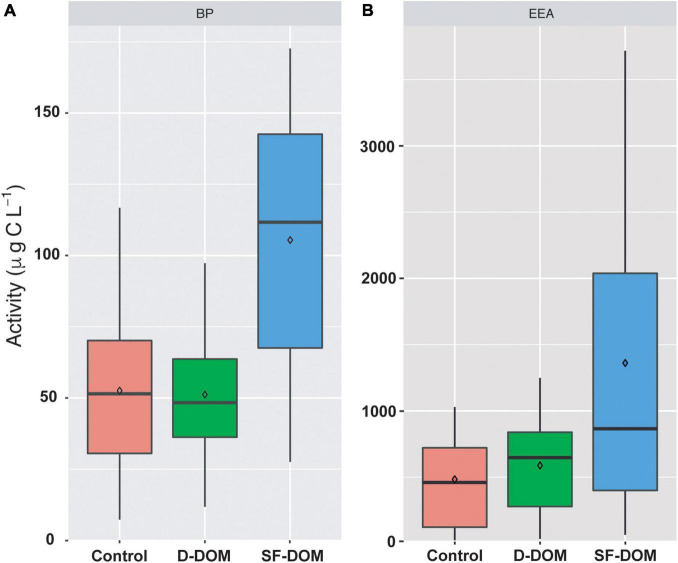
Time-integrated (96 h) rates of PB **(A)** and EEA **(B)** in controls and treatments of organic enrichment incubations from different periods and water depths of Puyuhuapi Fjord (*n* = 20). In the boxplots, the box indicates the interquartile ranges (25 and 75th percentiles), bold line into the box the medians, diamond the averages, and vertical lines outsides values.

**FIGURE 3:**
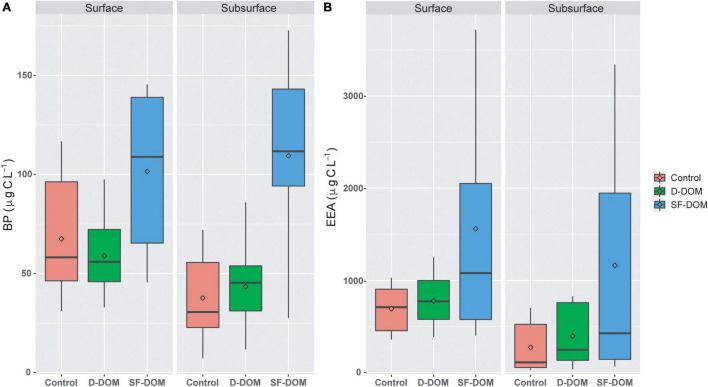
Time-integrated (96 h) rates of BP **(A)** and EEA **(B)** in controls and organic enrichment incubations of surface and subsurface waters from different experimental periods in Puyuhuapi Fjord (*n* = 10). Significant differences (Mann-Whitney, *p*-value < 0.05) were found between Control and SF-DOM and between D-DOM and SF-DOM for BP in surface and subsurface waters. In the boxplots, the box indicates the interquartile ranges (25 and 75th percentiles), bold line into the box the medians, diamond the averages, and vertical lines outsides values.

Time-integrated BP and EEA rates in SF-DOM treatment were significantly higher (Mann-Whitney, *p-value* < 0.05) than those estimated for controls when the data set was analyzed as a whole (Figure 2). In addition, PB rates estimated in SF-DOM were also significantly higher than in D-DOM treatment. Indeed, average BP and EEA in SF-DOM enrichment incubations increased by a factor of 2 compared to the rates measured in controls (Figure 2). In contrast, average rates of BP and EEA under D-DOM addition were not significantly different to those in controls (Mann-Whitney, *p-value* > 0.05) (Figure 2). Integrated BP rates for surface waters were significantly higher than in subsurface waters under control conditions (Mann-Whitney, *p-value* < 0.05), and rates of EEA for both D-DOM treatment and controls were significantly higher in surface than subsurface water incubations (Mann-Whitney, *p-value* < 0.05) when the data set was analyzed as a whole (Figure 3).

Time-integrated BP and EEA rates showed seasonality during the study period (Kruskal-Wallis, *p-value* < 0.05) (Supplementary Figure 3) with the highest values (BP = 134–173 μg C L^–1^; EEA = 1,827–3,717 μg C L^–1^) estimated in austral summer (March 2017 and February 2019) and the lowest (BP = 7–10 μg C L^–1^; EEA = 26–36 μg C L^–1^) in austral winter 2018 (July). During winter 2019 (July) integrated BP rates were also elevated (80–150 μg C L^–1^) but were accompanied by low rates of enzymatic hydrolysis (50–500 μg C L^–1^) (Supplementary Figure 3). Integrated BP rates in the SF-DOM treatment were significantly higher (Mann-Whitney, *p-value* < 0.05) than those measured in D-DOM and control treatments during most of the studied periods. For EEA integrated rates were significantly higher during austral summer (March 2017 and February 2019). Maximum rates of integrated BP (171.2 ± 2.1 μg C L^–1^) recorded in the SF-DOM treatment in subsurface water from austral summer (February 2019) coincided with high rates of integrated EEA (3,224 ± 166 μg C L^–1^). In contrast, in subsurface water incubations of austral winter (July 2018) the lowest rates of integrated BP (<30 μg C L^–1^) and EEA (<100 μg C L^–1^) were detected (Supplementary Figure 3).

### Bacterioplankton Production and Extracellular Enzymatic Activity in Meltwater Incubations in Proglacial Fjords

**FIGURE 4:**
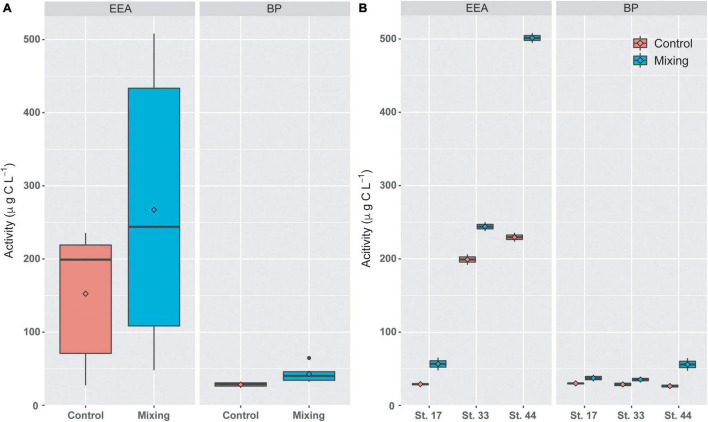
Time-integrated (72 h) rates of EEA and BP in controls and mixing treatments of the combined (**(A)**, *n* = 6) and individual stations (**(B)**, *n* = 2) from the proglacial fjords area of the Southern Patagonian Icefield. Stations 17 and 33 are under higher influence of meltwaters, whereas Station 44 was under the influence of oceanic waters. In the boxplots, the box indicates the interquartile range (25 and 75th percentiles), bold line into the box the median, diamond the averages, and vertical lines outsides values.

Time-integrated rates of BP and EEA were higher in mixing treatment than in controls, with significant differences observed in BP (Mann-Whitney, *p-value* < 0.05) when the whole data set was analyzed (Figure 4A). When stations were treated separately, rates of BP and EEA estimated at Station 44 from the mixing treatment (501.4 ± 9.5 and 56 ± 12.3 μg C L^–1^, respectively) were higher than those measured at stations 17 and 33 (Figure 4B). Additionally, integrated BP and EEA at stations 17 and 33 were similar between controls and treatments. In contrast, at station 44 rates of BP and EEA in the mixing treatment were higher than those measured under control conditions (Figure 4B). These comparisons were supported by significant differences (Mann-Whitney, *p-value* < 0.05) observed in discrete rates of BP and EEA.

### Bacterioplankton Abundance and Growth Efficiency in Dissolved Organic Matter Incubations

Bacterioplankton abundance (BA) increased from t0 to tf during incubations, showing highest average values in SF-DOM treatments of surface (7,105 ± 800 × 10^6^ cells L^–1^) and subsurface (4,648 ± 291 × 10^6^ cells L^–1^) waters during February 2019 (Table 2 and Supplementary Figure 4). In contrast, the lowest average values (<1,000 × 10^6^ cells L^–1^) were recorded in subsurface water during winter experiments (July 2018 and July 2019) (Table 2 and Supplementary Figure 4). With the exception of February 2019, surface bacterioplankton abundances were significantly higher than those measured in subsurface waters (Mann-Whitney, *p-value* < 0.05), but did not show significant differences between controls and treatments (Kruskal-Wallis, *p-value* > 0.05), both in surface and subsurface waters (Supplementary Figure 4). In February 2019 (austral summer), abundances in the SF-DOM treatment of surface and subsurface waters were higher than those observed in control and D-DOM incubations during the last 48 h of the experiments (Supplementary Figure 4).

Bacterioplankton growth efficiency was significantly higher (Mann-Whitney, *p-value* < 0.05) during austral summer (37–79% in February 2019) than during austral winter (13–23% in July 2018) (Table 2). BGE in surface and subsurface waters incubations did not show significant differences between controls and treatments, either in summer or winter (Kruskal-Wallis, *p-value* > 0.05). However, higher BGE estimates were observed in the SF-DOM treatments during February 2019 (67–79%) relative to those estimated in the D-DOM (45–52%) and control treatments (37–46%) (Table 2).

### Bacterioplankton Community Diversity

**FIGURE 5:**
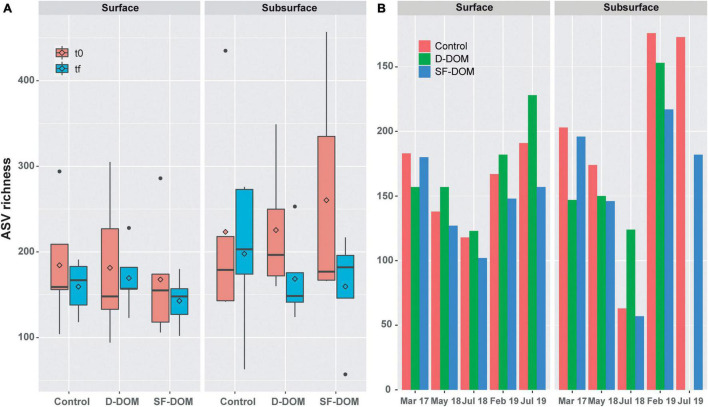
ASV richness of bacterioplankton at t0 and tf in controls and inorganic enrichment incubations of surface and subsurface waters in Puyuhuapi Fjord (**(A)**, *n* = 5). ASV richness at tf in controls and organic enrichment incubations of surface and subsurface waters **(B)**. No significant differences at 95% of confidence were observed between times of incubations and between control and treatments at tf.

Diversity of free-living (0.22 to 0.8 μm) bacterioplankton was analyzed by comparing a total of 8,549,865 16S rRNA gene sequences, which corresponds to 5,024 and 161 different ASVs of Bacteria and Archaea, respectively. Rarefaction curves reached the curvilinear phase of sampling effort, and most had started to plateau (Supplementary Figure 5). In the waters of Puyuhuapi Fjord, alpha diversity – measured as observed ASVs – ranged from 94 to 457 at t0, and between 57 and 276 at tf, with the lowest and highest average richness observed in subsurface waters in July 2018 at tf and in July 2019 at t0, respectively. A reduction in average ASV richness from t0 to tf was observed mostly in subsurface waters and in the SF-DOM treatments when the data set was analyzed as a whole (Figure 5A), however these changes were not significant at 95% of confidence. Among periods, a higher decreasing was observed in subsurface waters during austral winter (July 2018 and 2019; Supplementary Figure 6). Analyzing tf data alone, ASVs richness decreased in the SF-DOM treatments relative to controls in surface and subsurface waters during all periods assayed (Figure 5B). In contrast, with exception of March 2017, ASVs richness increased in the D-DOM treatment relative to controls during all periods in surface waters, as well as in July 2018 of subsurface waters incubations (Figure 5B).

**FIGURE 6:**
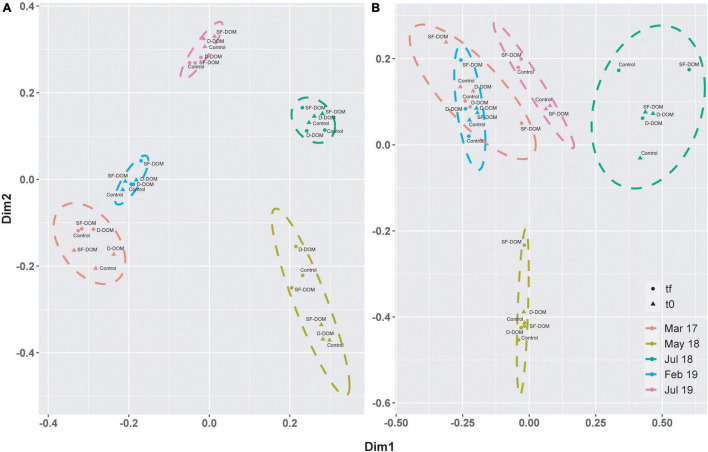
Principal coordinate analysis (PCoA) based on Bray-Curtis similarity analysis of free-living bacterioplankton communities in DOM incubations of surface **(A)** and subsurface **(B)** waters from Puyuhuapi Fjord. Colors represent experimental periods, and the ellipse shows the limits (at 95% confidence) of each period in the ordination space. Symbols indicate time of incubation (t0 and tf) and labels identify controls and type of treatment.

**FIGURE 7:**
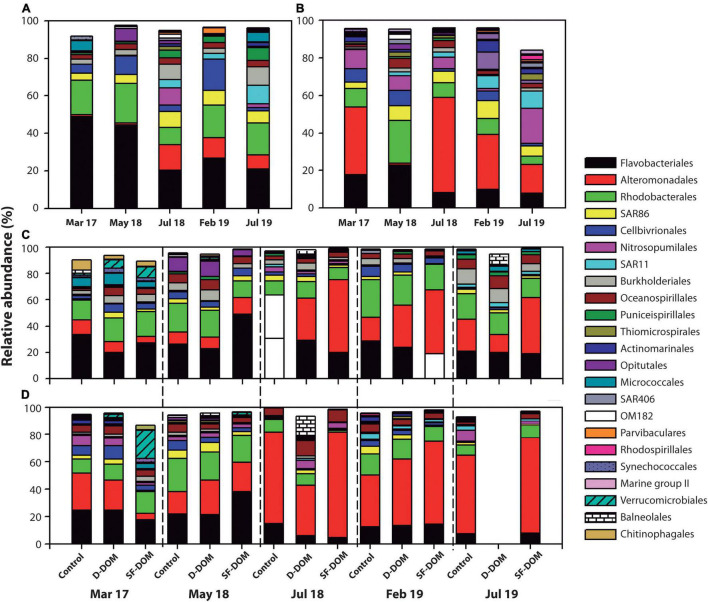
Relative abundance of predominant orders of free-living bacterioplankton at t0 in surface **(A)** and subsurface **(B)** waters during DOM microcosm incubations in Puyuhuapi Fjord. Contribution of predominant orders to composition of free-living bacterioplankton at tf in controls and DOM treatments of surface **(C)** and subsurface **(D)** waters.

Principal coordinate analysis (PcoA) showed strong differences in beta-diversity of bacterioplankton community of Puyuhuapi Fjord between periods in surface waters, whilst in subsurface waters the principal differences were observed for May and July 2018 (Figure 6). This analysis also highlighted differences in beta diversity between t0 and tf within some of the sampling periods (Figure 6). PERMANOVA analysis showed significant differences between compositions of prokaryotic communities among periods in surface (*p-value* < 0.05, Pseudo-F = 12.74) and subsurface waters (*p-value* = 0.05, Pseudo-F = 9.97). At the order level, *Flavobacteriales* and *Rhodobacterales* represented ca. 70% at t0 in surface waters from March 2017 and May 2018, whereas in February and July 2018, *Flavobacteriales*, *Alteromonadales*, *Rhodobacterales* and the SAR86 clade represented ∼ 50 to >60% of sequences recovered (Figure 7A). In incubations of subsurface waters at t0 (Figure 7B), members of *Alteromonadales* predominated the bacterioplankton community (∼30–50%) during March 2017, July 2018 and February 2019, while in May 2018 *Flavobacteriales* and *Rhodobacterales* contributed 23% each, and in July 2019 *Nitrosopumilales* and *Alteromonadales* represented 18 and 15% respectively (Figure 7B). At tf, changes in the contribution of representative orders relative to controls in surface waters were mostly observed in the treatment with SF-DOM and were characterized by increases in the relative proportion of *Flavobacteriales* in May 2018 and of *Alteromonadales* in July 2018, and February and July 2019 (Figure 7C). In subsurface waters, major changes were also observed in SF-DOM incubations, principally characterized by a decrease in the relative contribution of *Alteromonadales* and an increase in *Verrucomicrobiales* in March 2017, and increases in *Flavobacteriales* in May 2018 and *Alteromonadales* in July 2018, and February and July 2019 (Figure 7D).

**FIGURE 8:**
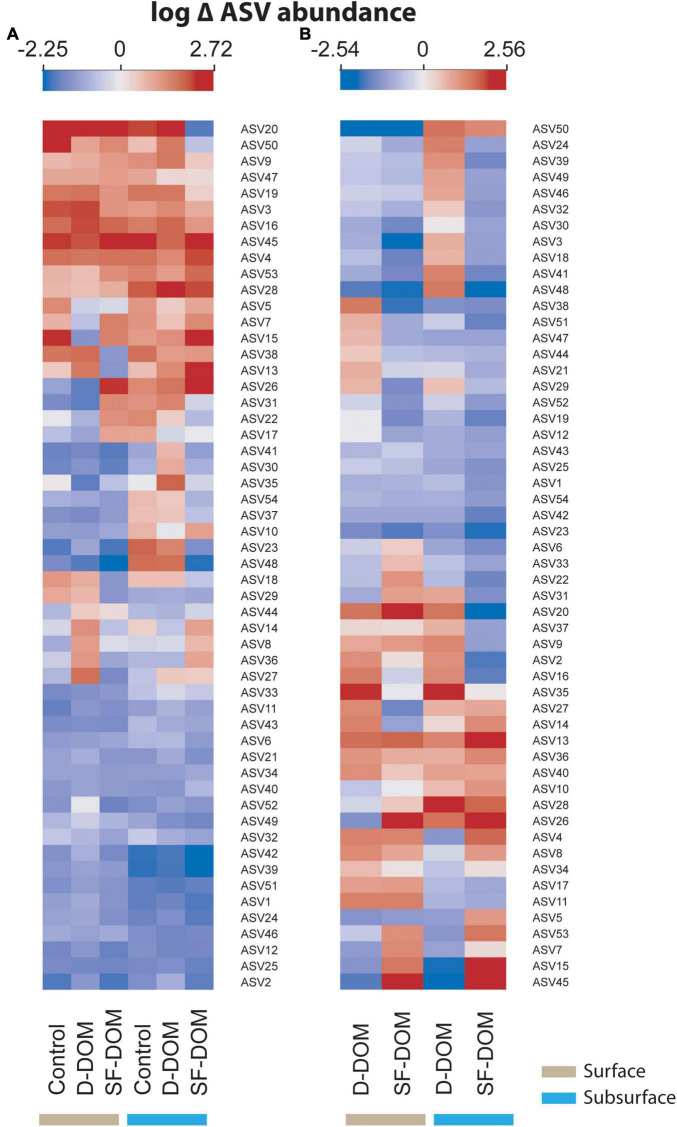
Heat maps showing average changes (log scale) in the abundance of representative ASVs of bacterioplankton in controls and DOM treatments between t0 to tf **(A)**, and in DOM treatments relative to controls at tf **(B)** for incubations in Puyuhuapi Fjord.

At the ASVs level, when the abundance from different periods was averaged, a significant proportion of the representative taxa showed decreases between t0 and tf in controls (65%), and in D-DOM and SF-DOM treatments (65 and 69%, respectively) in surface waters incubations (Figure 8A). For incubations of subsurface waters, 50% of representative ASVs increased in controls and D-DOM incubations, whereas 63% decreased in the treatment with SF-DOM (Figure 8A). Among representative taxa showing these decreased abundances, we identified bacterial ASVs belonging to the SAR11, SAR86 and SAR92 clades, uncultured *Flavobacteriales* of the groups NS4 and NS5, and the archaea *Candidatus Nitrosopumilus* (Figure 8A and Table 4). At the end of incubations (tf), 57 and 69% of representative ASVs showed reduced averaged abundance in SF-DOM treatments relative to controls, in surface and subsurface waters respectively (Figure 8B), whereas in D-DOM incubations reductions of between 50 and 56% of representative taxa were observed (Figure 8B). Among ASVs that reacted positively to the addition of D-DOM (∼18%), several genera belonged to *Gammaproteobacteria*. For SF-DOM, a higher proportion of representative taxa showed increases in surface (>50%) than in subsurface water (ca. 30%) incubations, and of these, several members belonged to the groups *Flavobacteriales* and *Alteromonadales* (Figure 8B and Table 4). Differences in the response of ASVs abundances to the addition of D-DOM and SF-DOM relative to the controls were more evident in subsurface waters of March 2017, July 2018 and February 2019 and in surface waters of May 2018 and July 2019 (Supplementary Figure 7). Opposite responses to the addition of D-DOM vs. SF-DOM relative to the controls were mostly associated with members of the family *Flavobacteriaceae* (e.g., NS3 and NS5 clade, *Ulvibacter* sp., *Polaribacter* sp.) (Supplementary Figure 7 and Table 4).

**TABLE 4:** Taxonomic assignment for main representative ASVs (ASVs containing more than 1000 sequences) identified in microcosm incubations with waters of Puyuhuapi Fjord and proglacial fjords.

ASV ID	Taxonomic assignment	ASV ID	Taxonomic assignment
ASV1	*Nitrosopumilales; Candidatus_Nitrosopumilus*	ASV37	*Flavobacteriales; Flavobacteriaceae; Formosa*
ASV2	*SAR86*	ASV38	*Rhodobacterales; Rhodobacteraceae; Planktomarina*
ASV3	*Burkholderiales; Comamonadaceae; RS62*	ASV39	*Thiomicrospirales; Thioglobaceae; SUP05*
ASV4	*Alteromonadales; Pseudoalteromonadaceae; Pseudoalteromonas*	ASV40	*Burkholderiales; Methylophilaceae; OM43*
ASV5	*Flavobacteriales; Flavobacteriaceae; NS3a*	ASV41	*Cellvibrionales; Porticoccaceae; SAR92*
ASV6	*Cellvibrionales; Porticoccaceae; SAR92*	ASV42	*Nitrosopumilales; Nitrosopumilaceae; Candidatus_Nitrosopumilus*
ASV7	*Flavobacteriales; Flavobacteriaceae; Polaribacter*	ASV43	*Cellvibrionales; Porticoccaceae; SAR92*
ASV8	*Burkholderiales; Methylophilaceae; OM43*	ASV44	*Flavobacteriales; Flavobacteriaceae; NS5*
ASV9	*Cellvibrionales; Porticoccaceae; SAR92*	ASV45	*Alteromonadales; Colwelliaceae; Colwellia*
ASV10	*Flavobacteriales; Flavobacteriaceae;*	ASV46	*Flavobacteriales; Flavobacteriaceae; NS5*
ASV11	*Flavobacteriales; Flavobacteriaceae; NS5*	ASV47	*Flavobacteriales; Flavobacteriaceae; Ulvibacter*
ASV12	*SAR11; Clade_Ia*	ASV48	*Rhodobacterales; Rhodobacteraceae; Amylibacter*
ASV13	*Verrucomicrobiales; Rubritaleaceae; Persicirhabdus*	ASV49	*Nitrosopumilales; Nitrosopumilaceae;*
ASV14	*Flavobacteriales; Flavobacteriaceae; uncultured*	ASV50	*Flavobacteriales; Flavobacteriaceae; Polaribacter*
ASV15	*Alteromonadales; Pseudoalteromonadaceae; Pseudoalteromonas*	ASV51	*Actinomarinales; Actinomarinaceae; Candidatus_Actinomarina*
ASV16	*Oceanospirillales; Pseudohongiellaceae; Pseudohongiella*	ASV52	*SAR86*
ASV17	*Flavobacteriales; Flavobacteriaceae; Formosa*	ASV53	*Oceanospirillales; Marinomonadaceae; Marinomonas*
ASV18	*Rhodobacterales; Rhodobacteraceae;*	ASV54	*Cellvibrionales; Halieaceae; OM60 (NOR5)*
ASV19	*Alteromonadales; Colwelliaceae; Colwellia*	ASV55	*Flavobacteriales; Flavobacteriaceae; NS5 marine group*
ASV20	*Alteromonadales; Alteromonadaceae; Glaciecola*	ASV56	*Flavobacteriales; Flavobacteriaceae; Flavobacterium*
ASV21	*SAR11; Clade_II*	ASV57	*Alteromonadales; Colwelliaceae; Colwellia*
ASV22	*Flavobacteriales; Flavobacteriaceae; Tenacibaculum*	ASV58	*Rhodobacterales; Rhodobacteraceae; uncultured*
ASV23	*Flavobacteriales; Flavobacteriaceae; NS3a*	ASV59	*Oceanospirillales; Marinomonadaceae; Marinomonas*
ASV24	*SAR86*	ASV60	*Vibrionales; Vibrionaceae;*
ASV25	*SAR11; Clade_Ia*	ASV61	*Oceanospirillales; Marinomonadaceae; Marinomonas*
ASV26	*Flavobacteriales; Flavobacteriaceae; Polaribacter*	ASV62	*Pseudomonadales; Moraxellaceae; Psychrobacter*
ASV27	*Actinobacteria; Micrococcales; Microbacteriaceae; Candidatus_Aquiluna*	ASV63	*Rhodobacterales; Rhodobacteraceae;*
ASV28	*Alteromonadales; Colwelliaceae; Colwellia*	ASV64	*Oceanospirillales; Saccharospirillaceae; Thalassolituus*
ASV29	*Opitutales; Puniceicoccaceae; Lentimonas*	ASV65	*Alteromonadales; Colwelliaceae; Colwellia*
ASV30	*Puniceispirillales; SAR116; Candidatus_Puniceispirillum*	ASV66	*Oceanospirillales; Saccharospirillaceae; Oleispira*
ASV31	*Flavobacteriales; Flavobacteriaceae; Ulvibacter*	ASV67	*Oceanospirillales; Saccharospirillaceae; Oleispira*
ASV32	*Flavobacteriales; Flavobacteriaceae; NS4*	ASV68	*Alteromonadales; Alteromonadaceae; Alteromonas*
ASV33	*Flavobacteriales; Cryomorphaceae; uncultured*	ASV69	*Alteromonadales; Colwelliaceae; Colwellia*
ASV34	*Cellvibrionales; Halieaceae; OM60 (NOR5)*	ASV70	*Cellvibrionales; Porticoccaceae; SAR92 clade*
ASV35	*Bacteroidota; Rhodothermia; Balneolales; Balneolaceae; Balneola*	ASV71	*Oceanospirillales; Saccharospirillaceae; Oleispira*
ASV36	*Synechococcales; Cyanobiaceae; Synechococcus_CC9902*		

**FIGURE 9:**
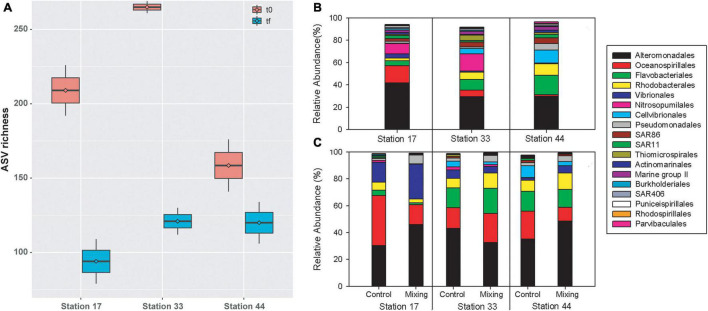
ASV richness in controls and treatments of free-living bacterioplankton at t0 and tf in water incubations at stations in the proglacial fjords region **(A)**, and community composition in controls at t0 **(B)** and in controls and treatments at tf **(C)**.

In the microcosms of glacial fjords waters, ASVs richness ranged between 141 (Station 44) and 269 (Station 33) at t0, and from 79 (Station 17) to 134 (Station 44) at tf. When averaged between controls and treatments, ASVs richness decreased from t0 to tf for all three stations, with greatest reduction observed at station 33 (144 ASVs) and the smallest reduction in Station 44 (39 ASVs; Figure 9A). At tf, a reduction in ASVs in the mixing treatment relative to control was observed at stations 17 and 44 (ca. 30 ASVs), whereas at station 33 richness increased in almost 20 ASVs. At the order level, composition of bacterioplankton was characterized by a predominance of sequences of *Alteromonadales*, which represented more than 40% of the relative abundance at Station 17 and ca. 30% at Stations 33 and 44 (Figure 9B). A significant proportion of *Flavobacteriales* (17%) was also observed at Station 44, followed by *Cellvibrionales* (ca. 12%) and *Rhodobacterales* (10%). At stations 17 and 33, archaea of the order *Nitrosopumilale*s represented 9 and 15%, *Oceanospirillales* 15 and 6%, *Flavobacteriales* 4 and 9% and *Rhodobacterales* 2 and 6%, respectively (Figure 9B). At tf, the observed changes in the mixing treatment relative to the control were represented principally by increases in the relative abundance of *Alteromonadales*, *Vibrionales*, and *Pseudomonadales* in Station 17 and 44 (Figure 9C), and in *Oceanospirillales*, *Rhodobacterales*, and *Pseudomonadales* at Station 33 (Figure 9C).

**FIGURE 10:**
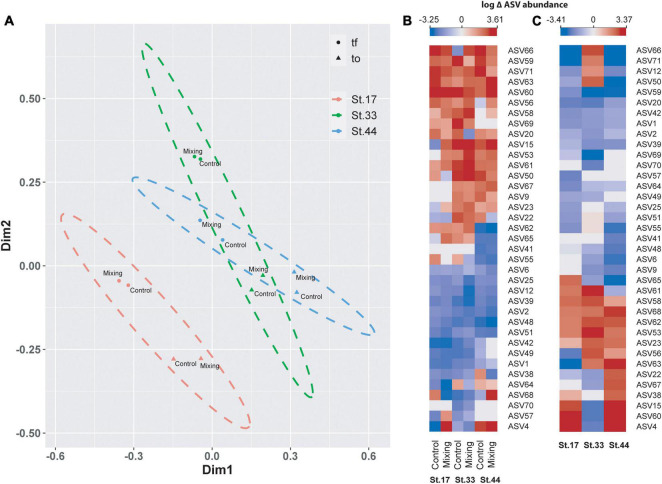
Principal coordinate analysis (PCoA) based on Bray-Curtis similarity analysis of free-living bacterioplankton communities in meltwater incubations **(A)** at stations in the proglacial fjords region. Colors represent stations, with the ellipse denoting limits (at 95% confidence) of each station within the ordination space. Symbols indicate time of incubation (t0 and tf) and individual labels identify controls and treatments. Heat maps showing changes (log scale) in the abundance of representative ASVs of bacterioplankton in controls and mixing treatments between t0 and tf **(B)**, and in mixing treatments relative to controls at tf **(C)** for each station.

Principal coordinate analysis highlighted differences in community composition of bacterioplankton between Station 17 and Stations 33 and 44 (PERMANOVA, Pseudo-F = 3.6 and 4.0, *p-value* < 0.05), and between t0 and tf within stations (Figure 10A). Among representative ASVs, between 38 (control at Station 17) and 54% (control at Station 33) showed increased abundance of sequences between t0 and tf, with between 38 and 49% in controls and treatments showing decreases (Figure 10B). Increases were shown by several members of the families *Saccharospirillaceae* (e.g., *Oleispira* sp.) and *Marinomonadaceae* (*Marinomonas* sp.) in the order *Oceanospirillales*, and *Flavobacteriaceae*, *Colwelliaceae* and *Rhodobacteraceae* (Figure 10B and Table 4). Decreased abundances of representative ASVs between t0 and tf were mostly observed in members of the archaea family *Nitrosopumilaceae* (e.g., *Candidatus Nitrosopumilus* and *Candidatus Actinomarina*), the SAR11 and SAR86 clades, and *Rhodobacteraceae* (*Amylibacter* and *Planktomarina* sp.) (Figure 10B and Table 4). At tf, 30–38% of representative ASVs increased in the mixing treatment relative to control (Figure 10C), with *Oleispira* sp., *Polaribacter* sp. and a member of the SAR11 clade showing significant increases in abundance only at Station 33. Members of the genera *Tenacibaculum* (*Flavobacteriaceae*) and *Oleispira* increased relative to control at Station 44, and *Pseudoalteromonas sp*., *Planktomarina* sp. and an unidentified member of *Vibrionaceae* showing increases relative to controls at stations 17 and 44 (Figure 10C and Table 4). Members of the genera *Alteromonas*, *Psychrobacter*, and *Marinomonas*, the NS3 marine clade (*Flavobacteriaceae*) and an uncultured genus of *Rhodobacteriaceae* all showed significant increases relative to controls in the three stations (Figure 10C and Table 4).

### Dissolved Organic Carbon, Inorganic Nutrients and Oxygen During Incubations

In the microcosm incubations in Puyuhuapi Fjord, DOC concentrations at t0 were significantly higher in treatments than in controls (Kruskal-Wallis, *p-value* < 0.05), in both surface and subsurface waters incubations (Supplementary Figure 8 and Table 2). DOC decreased significantly between t0 and tf (Mann-Whitney, *p-value* < 0.05) both in controls and in treatments of surface and subsurface waters (Supplementary Figure 8 and Table 2). DOC consumption, estimated as the difference between concentrations at tf and t0, was in most cases >40% (Table 2). In the microcosms in glacial fjords, DOC concentrations at t0 were higher at station 44 (∼200 μM) than at stations 17 and 33 (∼100 μM), in both controls and mixing treatments (Table 2). DOC consumption was less than 40% (Table 2) at all 3 sampling stations, both in control and mixing treatments (Table 2). The decrease in DOC between t0 and tf was significant only at station 44 (Mann-Whitney, *p-value* < 0.05; Table 2).

Nitrate and phosphate concentrations, measured in February and July 2019, remained relatively constant from t0 to tf in most experiments (Table 2). Dissolved Oxygen (DO) concentrations measured in surface experiments during July 2018 and February 2019, decreased from t0 to tf in treatments and controls, however these differences were not significant (Mann-Whitney, *p-value* > 0.05). Oxygen consumption (ΔO_2_ concentration) were significantly higher in winter (July 2018) than in summer (February 2019) experiments (Mann-Whitney, *p-value* < 0.05) (Table 2), with average values 3 times higher during July 2018 (Table 2).

## Discussion

Rates of BP and EEA were clearly enhanced in treatments enriched with allochthonous SF-DOM, and this was accompanied by reductions in ASVs richness and in abundance of representative taxa of free-living bacterioplankton. These changes were evident for different periods and in surface and subsurface waters of Puyuhuapi Fjord. In contrast, the addition of autochthonous D-DOM resulted in smaller changes in community structure, with rates of BP and EEA similar to those measured in the controls. In the glacial fjord area, small changes in BP and EEA were accompanied by more pronounced reductions of ASVs richness and moderate changes in community composition of bacterioplankton in incubations from locations under strong influence of meltwaters. Our findings demonstrate that organic substrates derived from salmon food are highly reactive and promote enhanced rates of microbial activity and possible selective pressures on the diversity of the bacterial communities in Patagonian fjords. In the glacial fjord area, we demonstrated that meltwaters decrease species richness but do not have a major impact on heterotrophic activity, suggesting a community response to maintain microbial functioning. Patagonian fjord ecosystem is currently subjected to pressures from intensive activity of salmon farm industry and glacial retreat. Our study provides valuable information on the potential effects of these pressures on microbial dynamics. Modifying the activity and community structure of heterotrophic microbes could significantly affect the role of fjord environments in the cycling of organic matter and the burial of organic carbon (Smith et al., 2015; Bianchi et al., 2020).

### Heterotrophic Bacterial Activity on Autochthonous vs. Allochthonous Dissolved Organic Matter

Addition of SF-DOM resulted in mean estimated integrated rates of BP and EEA that were at least 2 times higher than those for D-DOM (Figure 2), with maximum differences observed in subsurface waters and during austral summer (e.g., March 2017 and February 2019; Supplementary Figure 3). Our estimates of BP and EEA with SF-DOM were in the upper range of reports for the highly productive coastal upwelling ecosystems off central Chile (Cuevas et al., 2004; Pantoja et al., 2011b) and were also 2 and 4 times higher than previous estimations of EEA (90.4 ± 47.8 nM h^–1^; Gutiérrez, unpublished data) and BP (0.3 ± 0.2 μg C L^–1^ h^–1^, Montero, unpublished data), respectively, in surface waters of Puyuhuapi fjords. These data strongly indicate that DOM derived from salmon food is quickly metabolized and thus may be considered an important and reactive substrate for bacterial heterotrophic activity in Patagonian fjords. High reactivity of these substrates was consistent with molar C/N ratios of the salmon food assayed in our experiments (C/N ratio = 7.8 ± 0.1) that are within the range of those reported for diatoms (5.1 – 13.3, Finkel et al., 2010) and are slightly higher than the C/N of the phytoplankton from the Puyuhuapi fjord (C/N = 7.1 ± 2, unpublished data) and that of planktonic organic matter (e.g., Redfield ratio C/N = 6.6, Redfield, 1958). High rates of EEA of aminopeptidase are also consistent with the significant proportion of proteins in salmon food (39–43% in food used in this study), which is in the upper range of that reported for plankton (20-40%; Lee, 1988) and exceeds the typical range for marine phytoplankton (12-35%; Brown, 1991). Indeed, prior studies have reported that proteinaceous material represents a major fraction of organic matter delivered from salmon farms and have suggested as a high value for heterotrophic activity (Nimptsch et al., 2015; Kamjunke et al., 2017). In the oceans, amino acids and proteins are considered labile substrates supporting a major proportion of microbial production (Nagata, 2008) and extracellular enzymatic hydrolysis (Hoppe et al., 2002), respectively. In fact, enhanced bacterial production, extracellular enzyme activity, cell abundance and growth of bacteria have been stimulated in enrichment experiments following addition of amino acids and proteins in the Baltic Sea (Pontiller et al., 2020). Excretion of phytoplankton is considered a significant source of dissolved nitrogen compounds (Granum et al., 2002; Nagao and Miyazaki, 2002), and in productive ecosystems the aminopeptidase activity is enhanced under phytoplankton bloom conditions (Gutiérrez et al., 2011; Pantoja et al., 2011b). The reactivities of organic substrates play a fundamental role in the dynamics and cycling of DOM, with labile compounds being more rapidly degraded than more recalcitrant ones (Koehler et al., 2012; Guillemette et al., 2013), and thus contributing to export refractory organics and carbon sequestration in deep waters by the microbial carbon pump (Jiao et al., 2010; Legendre et al., 2015). Here, we show enhanced heterotrophic activity associated with the surplus of an emerging “allochthonous” source of organic carbon in the coastal ocean. This finding challenges the traditional view of phytoplankton production as the primary source of labile DOM (del Giorgio and Davis, 2003) and the main support of microbial heterotrophic activity (Cole et al., 1982; Azam et al., 1983) for those areas that have a strong salmon farming activity.

The estimations of time-integrated rates of microbial activity showed that EEA processed on average 4.2 and 1.8 times more carbon than BP in surface and subsurface waters respectively, with the higher differences found in incubations with additions of SF-DOM (ca. 5-fold higher). Extracellular enzymatic hydrolysis plays a central role in the cleavage of macromolecules to smaller substrates suitable for microbial uptake (Hoppe et al., 2002; Arnosti, 2014), and therefore the coupling between the uptake and EEA is expected to influence the recycling and export of organic matter. We show here that a significant fraction of dissolved organic substrates delivered from the action of extracellular enzymes were not destinated to biomass production during incubations time. The EEA/BP ratio remained constant during the first 12 h of the incubations, as evidenced by the strong positive correlation between discrete rates of EEA and BP (Pearson coefficient *r* = 0.92, *p-value* < 0.000), indicating a coupling between substrate bioavailability and microbial production. After that, this relationship was weakened, as evidenced by the strong inverse correlation between the differences in EEA and BP rates between tf and t0, which was mostly driven by extreme changes (positives and negatives) resulting from the addition of SF-DOM (Pearson coefficient *r* = –0.82, *p-value* < 0.000; Supplementary Figure 9). We suggest that the addition of this “allochthonous” organic matter enhances the differences between the rates of EEA and BP and therefore influences the fate of organic matter delivered from enzymatic hydrolysis in Patagonian fjords. High EEA/BP ratios in surface waters, suggest both that a major fraction of organic substrates is fueling microbial respiration and/or can escape to bacterioplankton action and becomes available for carbon exporting. Based on our calculations of BGE for summer and winter 2019 (Table 2), we were able to estimate the bacterial carbon demand (BCD), and, contrary to that observed for BP rates, we found a positive correlation between the differences in EEA and BCD rates between tf and t0 during austral summer (Supplementary Figure 9). This result suggests that a significant fraction of organic substrates that were added and hydrolyzed by the action of extracellular enzymes during this period is channelized to bacterioplankton respiration, while in winter are likely supporting carbon exportation. In addition, estimates of EEA/BCD ratio of surface waters showed higher values in SF-DOM treatment (∼3) than in controls and D-DOM treatments (∼1.5). In contrast, EEA/BCD ratio showed values lower than one for controls and treatments in subsurface waters. We suggest that organic matter enrichment influences hydrolysis-uptake coupling in surface waters of Patagonian fjords and thus can promote carbon exportation. The carbon export to deeper waters can enhance the action of microbial carbon pump and fuel activity of subsurface heterotrophic microbes which promotes oxygen consumption. Consistently, some authors have proposed that the hypoxia observed in deep waters of Puyuhuapi Fjord could be partially explained by such organic matter respiration (Schneider et al., 2014; Silva and Vargas, 2014; Pérez-Santos, 2017).

High bacterioplankton activity on SF-DOM recorded during austral summer (February 2019) coincided with a significant decrease in DOC concentrations (45–54%), increased BA (14 times higher than at t0) and maximum BGE values (67–79%), suggesting that under summer conditions a significant fraction of the allochthonous DOM consumed can be efficiently transformed into bacterioplankton biomass and transferred via microbial loop into the pelagic food webs of Patagonian fjords. In contrast, a relatively lower BGE for the autochthonous D-DOM treatments and controls (45–52 and 37–46%, respectively) was accompanied by a moderate increase in BA (4 to 6 times higher than in t0) during the same period. During austral winter (July 2018), minimum BGE values (13–23%) and slight increases of BA (1 to 3 times higher than in t0) were estimated in both D-DOM and SF-DOM treatments and controls, indicating that a significant proportion of DOC consumed (44–66%) was respired by bacterioplankton. Although we did not directly determine respiration rates in our experiments, we observed higher oxygen decreases (Mann-Whitney, *p-value* < 0.05) during winter (ΔO_2_ = 0.94 ± 0.2 mL L^–1^) than summer incubations (ΔO_2_ = 0.3 ± 0.1 mL L^–1^). Our estimations of BGE were in the range (10–60%) of those previously reported for estuaries (del Giorgio and Cole, 1998 and references therein), but showed maximum values (>65%) with the addition of SF-DOM. Consistently, high BGE values (50–88%) have been observed in experiments with addition of highly labile substrates (Cuevas et al., 2011; Attermeyer et al., 2014). In aquatic ecosystems, BGE shows a wide range of spatial and temporal variability associated with changes in both abiotic and biotic factors (del Giorgio and Cole, 1998; Carlson et al., 2007), with seasonality being likely modulated by substrate availability (del Giorgio and Cole, 1998).

### Effect of Organic Matter Enrichment on Bacterioplankton Diversity

Our results suggest reduction of average ASVs richness during incubations most notably in subsurface waters and in the austral winter incubations (July 2018 and 2019), where ASVs richness decreased by ca. 50%. This suggests that bacterioplankton in subsurface waters in winter were more susceptible to modification of diversity under induced perturbations. In Puyuhuapi Fjord, reduction in ASVs richness of surface water prokaryotes has been associated with rising temperatures during warmer periods (Gutiérrez et al., 2018). In contrast, prokaryote richness in subsurface waters did not show notable temporal variability (Gutiérrez et al., 2018), which could be indicative that they are more resilient to variability in environmental conditions. Because incubation conditions remained constant during our experiments, observed changes in ASVs richness are likely to be associated with additions of organic substrates. Indeed, addition of SF-DOM resulted in decreased bacterioplankton richness relative to controls in all incubations, whereas addition of D-DOM promoted diversity in surface waters and moderately reduced diversity in subsurface waters (Figure 5). Similarly, SF-DOM treatments influenced community composition, evidenced by variations in the proportion of predominant taxa at the order level (Figure 7), and by decreased sequence abundances (at tf relative to t0 and controls) in a significant proportion of representative ASVs (Figure 8). Moreover, contrasting changes were detected in the abundance of a fraction of representative ASVs in the treatments with D-DOM and SF-DOM after incubations (Figure 8). These findings suggest that the organic matter released from salmon food during farming operations (e.g., unconsumed food) could be exerting a selective pressure on the diversity of microbial communities while increases rates of heterotrophic activity. Despite the differences observed in community composition among periods, especially in surface waters (Figures 6, 7), we observed recurrent changes for some taxonomic groups in response to substrate addition. Thus, representative ASVs that were markedly impacted by organic enrichments included members of the families *Flavobacteriaceae* and *Rhodobacteraceae*, taxa which are recognized as specialized degraders of phytodetritus that can respond rapidly to increased production of organic matter during bloom conditions (Buchan et al., 2014). Other families impacted include the *Colwelliaceae* with members considered to be ubiquitous aerobic organoheterotrophs (Stelling et al., 2014) also able to degrade hydrocarbons (e.g., Baelum et al., 2012; Mason et al., 2014), and the SAR86 clade which is an ubiquitous marine heterotroph (Hoarfrost et al., 2020). In aquatic ecosystems, increases in microbial diversity are closely linked to availability of substrates and microbial activity (Landa et al., 2014), and consequently with improved ecosystem functioning (Finlay et al., 1997). In contrast, the present study provides evidence of enhanced heterotrophic activity and suggest reduction in diversity induced by allochthonous input of highly reactive organic matter. These effects of organic enrichment could become critical in Patagonian fjords when combined with the climatically driven changes in water temperature and salinity that are also driving changes in microbial community diversity (Gutiérrez et al., 2015, 2018).

### Effect of Glacial Meltwaters on Activity and Diversity of Bacterioplankton

Changes in salinity (e.g., meltwater, river runoff) can influence growth and activity of bacterioplankton (del Giorgio and Bouvier, 2002; Lindh et al., 2015) and the structure of bacterial communities (Piquet et al., 2010; Fortunato and Crump, 2011; Herlemann et al., 2011; Campbell and Kirchman, 2013; Signori et al., 2014; Gutiérrez et al., 2015). In our transplant experiments, where bacterial communities from subsurface saline waters were incubated in surface meltwaters, time-integrated BP and EEA showed slight to moderate increase relative to controls. This suggests a functional adaptability of subsurface bacterioplankton community to the freshwater runoff associated with glacial melting. However, sites with greater glacial influence, i.e., higher salinity differences in mixing treatments (St. 17, ΔS = 17.4; St. 33, ΔS = 8.8), showed weaker increases in BP and EEA during the incubation period than the station with a lower influence of meltwaters (St. 44, ΔS = 2.2). These variations were inversely related to changes in bacterioplankton diversity during incubations (between t0 and tf), with greater reduction of ASVs richness in stations most strongly influenced by glacial melting (Figures 4B, 9A). These data agree with previous studies showing changes in environmental microbial diversity both in glacial fjords of Patagonia (Gutiérrez et al., 2015) and in other environments influenced by meltwaters (Zeng et al., 2009; Piquet et al., 2010, 2011). Thus, our findings showing changes in diversity that are not accompanied by variations in functional capabilities suggests that microorganisms can adjust community structure to sustain heterotrophic activity in fjords most strongly influenced by glacial melting. Several Patagonian glaciers are showing progressive retreat (Willis et al., 2012), however the influence of meltwaters on glacial fjords is a dynamic process that depends on local and regional climatic and oceanographic variability at different scales (Rivera et al., 2012; Moffat, 2014). Thus, short term variability (e.g., seasonal) in both melting and hydrodynamics in Patagonian proglacial fjords (e.g., Rivera et al., 2012; Ross et al., 2015) could promote functional adaptability in microbial communities to overcome the effect of freshening in fjord waters.

### Influence of Salmon Farming Activities on Bacterioplankton Processes in Puyuhuapi Fjord

Between 3–5% of the total food supplied to farmed salmon is estimated to be uneaten (Reid et al., 2009; Wang et al., 2012; Yoshikawa and Eguchi, 2013), and thus considered a major source of waste (Reid et al., 2009). In Puyuhuapi Fjord, a total of 18 farms have reported ca. 50,000 tons of salmon production during 2018 (SERNAPESCA, 2018). With average values for feed conversion factor of 1.25%, and 5% for feed loss (Yoshikawa and Eguchi, 2013), we estimate that in each farm 3,472 tons of food are used to sustain salmon production, and 173.6 tons lost as uneaten food. The carbon content of salmon feed pellets has been estimated at ∼50%, and of this ∼15% can be transferred to the dissolved fraction (Wang et al., 2012; Yoshikawa and Eguchi, 2013). Assuming a standard size for salmon cage of 20 m × 20 m × 30 m, an average of 17 floating cages per farm (Elizondo-Patrone et al., 2015) and an arbitrary average depth of 50 m for typical farm locations in Patagonia, we estimated ca. 9 g DOC m^–2^ d^–1^ being added by one salmon farm into the waters of the Puyuhuapi Fjord. At the scale of one farm, this DOC derived from salmon food represents 3–4 times the daily carbon synthesized by primary producers during typical productive pulses in this fjord (2–3 g C m^–2^ d^–1^; Montero et al., 2017a) and is 15 times higher than the carbon processed by BP (0.6 g C m^–2^ d^–1^; Daneri unpublished data) in this area. Using our estimates of BGE associated with the allochthonous enrichment incubations, we calculated theoretical rates of bacterial respiration of 4 and 7 g C m^–2^ d^–1^ during summer and winter, respectively. These values exceed – by a factor of 2 – the maximum rates of community respiration (2–3.5 g C m^–2^ d^–1^) estimated for Puyuhuapi Fjord (Montero et al., 2017a), indicating that under a scenario of elevated salmon production, heterotrophic processes would become predominant. Under a hypothetical condition of minimum water ventilation, dissolved oxygen could therefore be depleted over time periods of 12- and 24-days during winter and summer conditions, respectively. This theoretical exercise makes several strong assumptions; for example, we do not include oxygen supply and dilution of DOC through water circulation and mixing. On the other hand, we also do not include additional sources of organic matter that could exacerbate oxygen depletion, both from salmon farm activity (e.g., mortality, feces) and local coastal populations. We also do not consider hypoxia as a known recurrent condition already in waters of Puyuhuapi Fjord (Silva and Vargas, 2014; Pérez-Santos, 2017), a factor that could intensify our estimated impacts of salmon farm organic enrichment.

In summary, our study highlights salmon food-derived organic matter as an emerging substrate that enhances production and degradative capability of the free-living bacterioplankton in Patagonian fjords. Although not statistically conclusive our results also seem to indicate that salmon food-derived organic matter could be exerting selective pressure on microbial community diversity. We also provide evidence for a decoupling between the hydrolysis of organic macromolecules and the microbial uptake in presence of dissolved organic substrates released from salmon food: enzymatic hydrolysis processed up to 5 times more carbon than bacterioplankton production and was 3 times higher than estimated carbon demand. In aquatic ecosystems with high load of organic matter from different sources, such as Patagonian fjords (e.g., marine, terrigenous and human-derived organic substrates; González et al., 2019), a decoupling in the mechanisms controlling the efficiency of organic processing could have a significant effect on carbon cycling and nutrient remineralization, with consequences for the trophic status of these ecosystems. In the current scenario of strong human influences on coastal ecosystems, our findings open new questions on the preference of heterotrophic bacterioplankton for allochthonous rich organic matter over autochthonous substrates and the consequences for the recycling of organic carbon, carbon exportation, oxygen consumption and CO_2_ exchange in Patagonian fjords. The effect of these anthropogenic perturbations can be enhanced in proglacial fjords exposed to strong ice melting, where additional selective pressures on bacterioplankton diversity was evidenced.

## Data Availability Statement

The data presented in the study are deposited in the Sequence Read Archive (SRA) repository from NCBI (https://www.ncbi.nlm.nih.gov), accession numbers SRR15657559–SRR15657658.

## Author Contributions

PM and MG contributed to study design, data collection, analysis of data and interpretation of results, and manuscript leader. GD contributed to study design, critical revision and edition of final version of the manuscript. BJ contributed to data collection and analysis of results. All authors contributed to the writing of the manuscript.

## Conflict of Interest

The authors declare that the research was conducted in the absence of any commercial or financial relationships that could be construed as a potential conflict of interest.

## Publisher’s Note

All claims expressed in this article are solely those of the authors and do not necessarily represent those of their affiliated organizations, or those of the publisher, the editors and the reviewers. Any product that may be evaluated in this article, or claim that may be made by its manufacturer, is not guaranteed or endorsed by the publisher.
