# Supplementary material for: The Effect of Salmon Food-Derived DOM and Glacial Melting on Activity and Diversity of Free-Living Bacterioplankton in Chilean Patagonian Fjords
Source: Front Microbiol. 2022 Jan 11;12:772900. doi: 10.3389/fmicb.2021.772900 (PMC8787161; doi:10.3389/fmicb.2021.772900)
Supplement: Supplementary file 1 [file Data_Sheet_1.docx]

Supplementary Material

# Supplementary Figures and Tables

## Supplementary Figures





**Supplementary Figure 1.** Rates of BP during incubations of surface (A) and subsurface (B) waters of Puyuhuapi Fjord and in the stations of proglacial fjords area (C) of the Southern Patagonia Icefield. Error bars indicate standard deviation (n = 2).





**Supplementary Figure 2.** Rates of EEA during incubations of surface (A) and subsurface (B) waters of Puyuhuapi Fjord and in the stations of proglacial fjords area (C) of the Southern Patagonia Icefield. Error bars indicate standard deviation (n = 2).


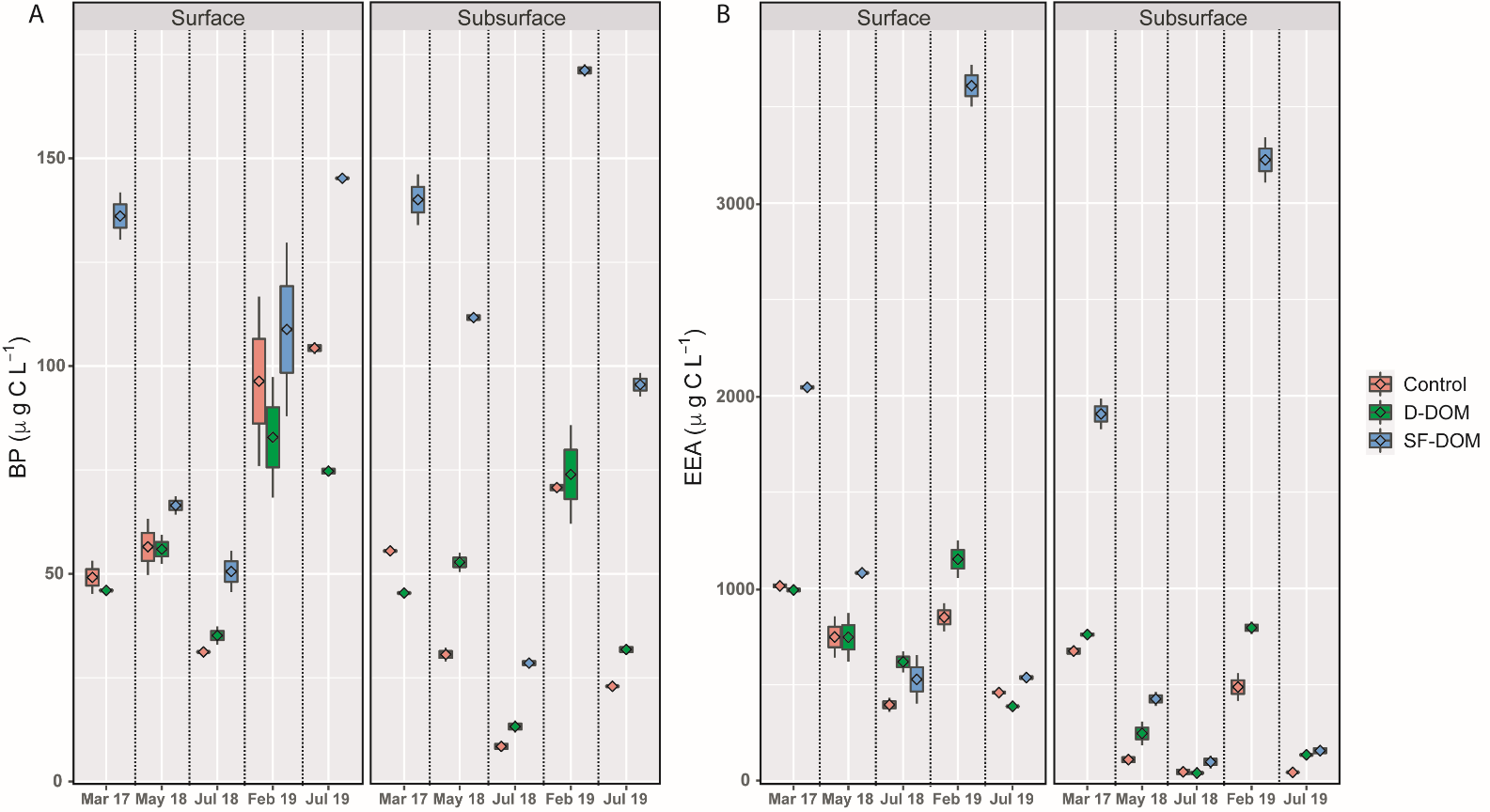


**Supplementary Figure 3.** Average time-integrated rates (96 hours, n = 2) of BP (A) and EEA (B) in control and treatments of DOM incubations of surface and subsurface waters during the experimental periods in Puyuhuapi fjord. In the boxplots, the box indicates the interquartile ranges (25 and 75^th^ percentiles), bold line into the box the medians, diamond the averages, and vertical lines outsides values.





**Supplementary Figure 4.** Bacterioplankton abundance (BA) during incubations of surface (A) and subsurface (B) waters of Puyuhuapi Fjord. Error bars indicate standard deviation (n = 2).

**Supplementary Figure 5.** Rarefaction curves for bacterioplankton assemblages from samples collected in waters of Puyuhuapi fjord and from the proglacial fjords area of the Southern Patagonia Icefield.


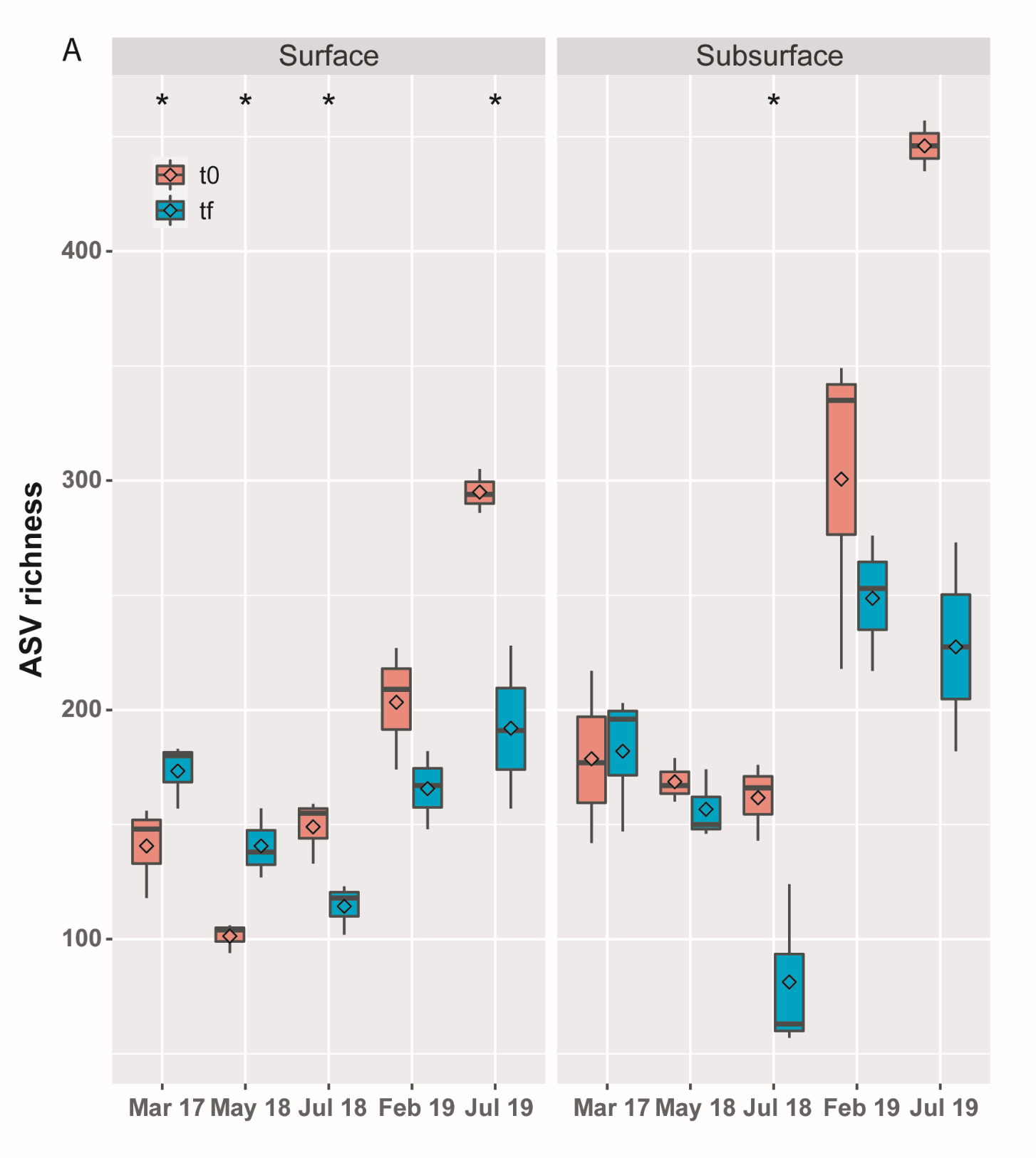


**Supplementary Figure 6.** ASV richness of the whole data (controls and treatments) at t0 and tf (for incubations of surface and subsurface waters of Puyuhuapi fjord in each experimental Period. The * indicate significant differences between t0 and tf at 90% of confidence.


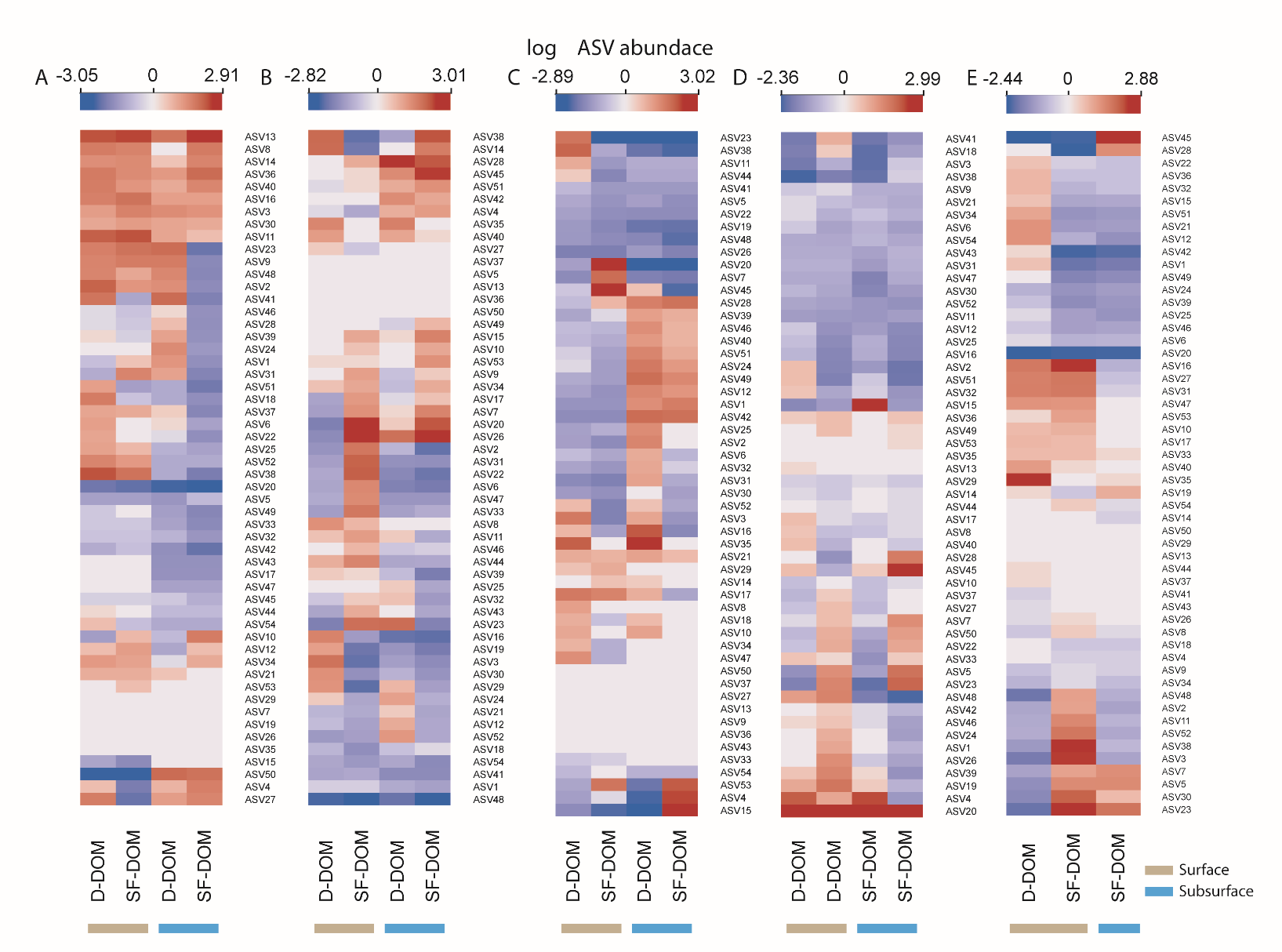


**Supplementary Figure 7.** Heatmaps showing changes (log scale) in the abundance of representative ASV of bacterioplankton in DOM treatments relative to controls at tf for incubations with waters of Puyuhuapi fjord during March 2017 (A), May 2018 (B), July 2018 (C), February 2019 (D) and July 2019 (E).

**Supplementary Figure 8.** Concentration of DOC in controls and DOM treatments at t0 and tf in incubations of surface and subsurface waters of Puyuhuapi fjord. Significant differences between t0 and tf (Mann-Whitney, p-value < 0.05) are indicated by *.

**Supplementary Figure 9.** Linear regression between the differences in EEA and BP rates between t0 to tf in controls and DOM treatments (A) and EEA and BCD rates between tf and t0 of controls and treatments during summer and winter (B) for incubations of Puyuhuapi fjord. Linear regressions were significant (*p-value* < 0.05) in A and for data from summer in B.
